# From Genotype to Phenotype: Through Chromatin

**DOI:** 10.3390/genes10020076

**Published:** 2019-01-23

**Authors:** Julia Romanowska, Anagha Joshi

**Affiliations:** 1 Department of Global Public Health and Primary Care, University of Bergen, 5018 Bergen, Norway; Julia.Romanowska@uib.no; 2 Computational Biology Unit, Department of Clinical Science, University of Bergen, 5021 Bergen, Norway

**Keywords:** epigenetics, chromatin modification, sequencing, regulatory genomics, disease variants

## Abstract

Advances in sequencing technologies have enabled the exploration of the genetic basis for several clinical disorders by allowing identification of causal mutations in rare genetic diseases. Sequencing technology has also facilitated genome-wide association studies to gather single nucleotide polymorphisms in common diseases including cancer and diabetes. Sequencing has therefore become common in the clinic for both prognostics and diagnostics. The success in follow-up steps, i.e., mapping mutations to causal genes and therapeutic targets to further the development of novel therapies, has nevertheless been very limited. This is because most mutations associated with diseases lie in inter-genic regions including the so-called regulatory genome. Additionally, no genetic causes are apparent for many diseases including neurodegenerative disorders. A complementary approach is therefore gaining interest, namely to focus on *epigenetic* control of the disease to generate more complete functional genomic maps. To this end, several recent studies have generated large-scale epigenetic datasets in a disease context to form a link between genotype and phenotype. We focus DNA methylation and important histone marks, where recent advances have been made thanks to technology improvements, cost effectiveness, and large meta-scale epigenome consortia efforts. We summarize recent studies unravelling the mechanistic understanding of epigenetic processes in disease development and progression. Moreover, we show how methodology advancements enable causal relationships to be established, and we pinpoint the most important issues to be addressed by future research.

## 1. Introduction

### 1.1. Definition of Epigenetics

**Figure 1:**
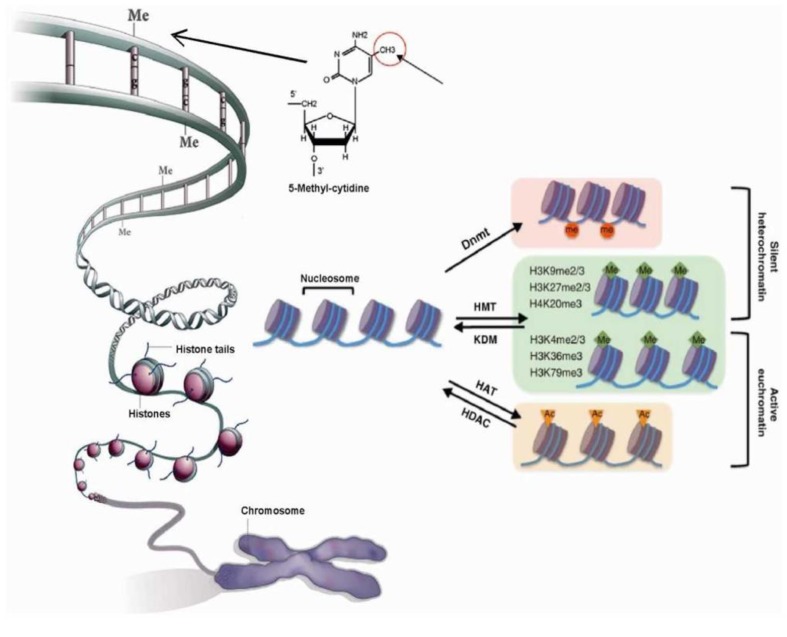
Diagrammatic representation of epigenetic mechanisms namely DNA methylation and chromatin modifications [14].

The human body consists of hundreds of different tissues and cell types, each with its characteristic well-defined function. For example, myosin is produced by muscle cells while hemoglobin is produced by red blood cells to facilitate cell type specific functions. Despite the diversity of functional molecules in an individual cell type, nearly all cell types in an organism contain the same genetic information or genome. To explain how this diversity of cell types can be achieved from a single cell or zygote, Conrad Waddington proposed the concept of “*epigenesis*” in 1956, where pluripotent cells have the “potential” to generate all other cell types of restricted potential, in which they gradually lose this “potential” during differentiation, famously depicted by the Waddington landscape [1]. This so-called potential was later associated with a physical phenomenon, the methylation of DNA [2], which is a methyl group added to position 5 on the cytosine ring. In mammals, it is mainly 5′—C—phosphate—G—3′ dinucleotide (CpG) that is subjected to methylation. Originally, methylation was found to act as a silencing mark. Accordingly, in embryonic stem cells, the majority of promoters have un-methylated DNA, and some of them become methylated during differentiation, assisting the acquisition of their final cell identity [3]. Over the years, many other epigenetic and transcription control mechanisms responsible for establishing unique gene expression profiles characteristic for different cell and tissue types during embryonic development have been studied in detail [4,5]. Gene regulatory elements receive and execute transcriptional signals, dependent on their epigenetic state and chromatin accessibility, controlling the expression of key developmental factors [6]. Chromatin dynamics are regulated through two main mechanisms: methylation of DNA and post-translational modifications of histone tails [7] (Figure 1). Histone modifications include, among others, phosphorylation, acetylation, methylation, and ubiquitylation, with methylation at specific residues as one of the most important posttranslational modifications regulating nuclear function, including transcriptional regulation, epigenetic inheritance, and maintenance of genome integrity [8]. Recently, it has become evident that histone modifications act together and a term “histone code” was coined to refer to a scheme of gene control exhibited by the complex interactions of histone modifications [9,10]. Accordingly, specific functions can be associated to a group of histone modifications, such as H3K27ac and H3K4me1, and are associated with enhancer regions. Several reviews written over the years focus on state-of-the-art studies providing structure function associations of histone modifications and successive layers of chromatin structure in mammalian genomes [11,12,13].

### 1.2. Broadening the Definition of Epigenetics

Epigenetics are widely understood as any mechanism by which heritable changes in gene expression occur without changing the DNA sequence, but the precise definition has evolved over the years. Apart from the above mechanisms, the role of non-coding RNAs (ncRNAs) is becoming evident in epigenetic control (reviewed in [15]). In short, ncRNAs are transcribed from the genome sequence without producing a functional protein, are highly cell type specific and regulate epigenetic patterning by establishing epigenetic modifications (DNA methylation and chromatin modifications). For example, *Xist* is an ncRNA expressed from the X chromosome that silences the other X chromosome in females. Non-coding RNAs can function as a guide or tethers, and may be the molecules of choice for epigenetic regulation of DNA methylation [16]. Some authors therefore now include ncRNAs in their definition of epigenetics. Nevertheless, we will stick to the classical definition and discuss only DNA methylation and chromatin modifications in this review.

### 1.3. Epigenetic Mechanisms Regulate Gene Expression Using Environmental Cues 

Epigenetic mechanisms are thought to act as a memory of a cell and might be the key process by which the environment interacts with the genome [17]. DNA methylation plays a crucial role during early development including active demethylation of paternal genome before the first cleavage and subsequent demethylation of maternal genome [18]. Furthermore, environmental factors also affect gene expression via epigenetic mechanisms during embryonic development, which can manifest into adulthood or even old age. Cigarette smoking is an environmental factor, associated with dose- and time-dependent changes in the DNA methylation signature, which manifests in gene and protein expression leading to an increased vulnerability to other forms of complex illnesses [19,20]. Harmful environmental factors need not be substances. Trauma and stress also influence gene expression through epigenetic mechanisms, and furthermore these epigenetic modifications can be passed over the generations [21].

## 2. Chromatin Modifications and the Genome Organization

### 2.1. Chromatin’s Structure Defines Its Function

To understand epigenetic control mechanisms, we will begin with the structure of chromatin. DNA is wrapped around the core histone proteins, forming a structure named nucleosome (two copies of H2A, H2B, H3, H4, and 147 base pairs (bps) of DNA around them). This is further compacted, with the assistance of assembly and packaging related proteins, to form a higher-order chromatin structure [22], with two distinct chromatin states “euchromatin” and “heterochromatin” (Figure 1). A more open chromatin environment, euchromatin, is where the majority of active genes localize, while heterochromatin is characterized by a more compact environment where inactive genes, non-coding DNA and repeat elements reside [8]. Heterochromatin can be further separated into two groups, facultative and constitutive. Facultative heterochromatin includes regions that consist of genes that are highly differentially expressed during development. Constitutive heterochromatin on the other hand is gene poor, rich in repeat elements, mainly found in centromeres and telomeres, and silenced indefinitely [23]. These chromatin states are marked by distinct epigenetic factors [24] (Figure 1), and in euchromatin, the histone modification density correlates with the density of TF binding sites [25]. However, neither euchromatin nor heterochromatin is marked uniformly with epigenetic and transcriptional signals. Chromatin is further organized into so-called topologically associated domains (TADs), (first described by Dixon et al. (2012) [26]), regions spanning several hundred kilobases. Topologically associated domains are organized hierarchically and are highly enriched for insulating factor CCCTC-binding factor (*CTCF)* binding and histone marks at the boundaries [27]. Intra-chromosomal interactions are particularly enriched within TADs and accordingly genes within a TAD show highly correlated gene expression. The chromatin structure allows manifestation of genetic information in a cellular context, and mutations in chromatin organization genes lead to developmental pathologies [28,29]. Understanding of cell type specific 3D genome organization is therefore highly valuable in a disease context [30,31], where by disruption of TADs can result in chromatin interaction changes leading to mis-regulation of oncogenic or tumor suppressor genes [32].

### 2.2. Chromatin Structure is Dynamic and Marked by Histone Modifications 

The chromatin structure is organized with the help of DNA sequence and epigenetic modifications, including histone modifications, and a cross-talk between them is potentially facilitated through histone amino (N)-terminal tails interacting with neighboring nucleosomes [33]. Of various histone modifications, the most well-studied types are methylation, acetylation, phosphorylation, and ubiquitination [8]. Histone modifications influence chromatin mainly in two ways. The first mode of the modifications affects directly the structure of the chromatin over a long or short distance by the recruitment of DNA binding proteins and chromatin remodelers affecting nucleosome location. Hence, nucleosome removal could open the chromatin and a possible transcription factor binding motif could be revealed, or otherwise, newly recruited nucleosomes could conceal a binding motif, hindering transcriptional machinery recruitment at the locus [34]. The second mode of histone modification is carried out by three sets of enzymes named “writer”, “reader”, and “eraser”, based on the function of each enzyme related to each histone modification. For example, COMPASS family members maintain H3K4m3 modification, while polycomb family members maintain H3K27me3 modification. Both activating (H3K4me3) and repressing (H3K27me3) modifications are indeed present simultaneously at promoters enriched for developmental genes and have a distinct sequence signature [35]. Histone modifications also work jointly with DNA methylation, for repression of gene loci [36].

## 3. Epigenetics in Disease Context

### 3.1. Genome-Wide Studies Are Not Enough

Monogenic diseases are caused by the malfunctioning of only a single gene. For example, fragile x syndrome is caused by epigenetic changes in the *FMR1* gene. The silenced promoter of *FMR1* in disease shows heterochromatin markers, including DNA hypermethylation and histone deacetylation. This can be treated by pharmacological reactivation of gene transcription, particularly through the use of DNA demethylating agents or inhibitors of histone deacetylases [37]. Unfortunately, the vast majority of common diseases are not caused by mutations in a single gene, but rather by a large number of single nucleotide variations (SNPs) spread throughout the genome. These diseases are therefore called complex diseases. Complex diseases including cancer, diabetes, and neurodegenerative disorders such as Alzheimer’s and Parkinson’s disease are common and therefore form a global health burden. Though a large number of genetic variants have been identified (and will be identified) that increase the risk for these diseases, most explain only a small fraction of risk. Moreover, despite the fact that over 1000 genetic loci are associated with susceptibility to common diseases in human [38], only a handful of these loci have resulted in the identification of causal genes or pathways for potential therapeutic applications [39]. It is becoming clear that understanding of only genetic variation will not be sufficient to get a complete understanding of disease, and the role of epigenetic alterations in gene regulation is becoming evident in many diseases, including cancer. Understanding how a genotype influences human health and disease now requires characterization of the epigenome as well. For example, copy number aberrations of genes responsible for writing, reading, and removing H3K9 methylation were identified in medulloblastoma, demonstrating that defective control of the histone code contributes to the pathogenesis of medulloblastoma [40]. Large studies have therefore been designed to unravel epigenetic malfunctionalities in diverse diseases (Table 1). It is important to note that another major challenge in interpreting genome-wide data in a clinical context is the fact that the vast majority of genetic and epigenetic modifications lie in non-coding genomic regions, particularly [41] where the disease-associated variants in enhancers explain a greater proportion of the disease heritability [42].

### 3.2. Largescale Epigenetic Studies in Cancer

#### 3.2.1. Epigenetic Mechanisms Are Major Drivers in Cancer

The studies exploring mutational landscapes of cancer have highlighted frequent mutations in genes encoding chromatin-associated proteins. The exploration of functional mechanisms behind these mutations have improved our understanding of oncogenic mechanisms at different levels of chromatin organization and regulation (reviewed in Valencia et al. (2019) [54]). DNA methylation remains by far the most studied epigenetic mechanism in cancer where inactivation of tumor-suppressor genes occurs as a consequence of hypermethylation of the gene promoters. Numerous studies have identified a broad range of genes silenced by DNA methylation in different cancer types [55]. Importantly, different cancer subtypes show characteristic DNA methylation signatures [56], which can be translated in clinical medicine by using hypermethylated promoters as biomarkers. Human pluripotent stem cells were found to have more hypermethylated DNA than fibroblast cells [57]. Similarly, oncogenesis is thought to modify the cell state into a stem or progenitor epigenetic state. In cancer, mutations in key transcription factors lead to changes in DNA methylation, such that the number of genes with gene expression changes explained by DNA methylation are 10-fold higher than those explained by genetic mutations. Over 75% of DNA hypermethylated genes are marked by polycomb repressor components forming bivalent chromatin [58]. Wang et al. [59] pointed to one molecular mechanism to explain the role of *MLL3* mutations in cancer pathogenesis by examining changes in histone modification and gene expression after depletion of Polycomb or COMPASS family members. Next, they proposed a potential therapeutic strategy for cancers harboring COMPASS mutations which will allow resetting the epigenetically (Polycomb/COMPASS) balanced state of gene expression.

#### 3.2.2. Epigenetic Mechanisms in Hematopoietic Malignancies and Their Therapeutic Implications

Epigenetic changes in cancer are possibly reversible making them precious targets for cancer therapy. Indeed, DNA methylation biomarkers with diagnostic, prognostic, and predictive power are already in clinical trials or in a clinical setting [60]. DNA methyltransferase inhibitors have been approved for the treatment of several hematopoietic malignancies, including myelodysplastic syndromes, chronic myelomonocytic leukemia, and acute myelogenous leukemia (AML) [61]. Other epigenetic regulatory mechanisms also play a critical role in the pathogenesis of AML. Epigenome-wide analyses of histone H3 acetylation identified that epigenetic silencing of PRDX2, a growth suppressor, contributed to the malignant phenotype in AML [48]. A combination of the H3K9me3 signature with established clinical prognostic markers outperformed prognosis prediction based on clinical parameters alone in AML [46]. Epigenetic control is systematically studied in other hematopoietic malignancies as well. For example, the translocation t (15;17) forming a chimeric PML–RARα transcription factor is the initiating event of acute promyelocytic leukemia. PML-RARα regulates key cancer related genes and pathways by inducing a repressed chromatin at its target genes [49]. The PML–RARα binding universally led to histone deacetylase (HDAC) recruitment, loss of histone H3 acetylation, and increased H3K9me3. Accordingly, several anticancer drugs acting as inhibitors of *HDAC* or bromodomain and extra-terminal proteins (*BET*) were designed, tested, and in clinical trial. The use of these inhibitors is not limited to hematopoietic malignancies. The *HDAC* inhibitors have been used in glioblastomas, where mutations in tumor suppressors such as *IDH1* induce epigenetic changes that drive the development of gliomas [47]. Both *HDAC* and *BET* inhibitors work synergistically, primarily by suppressing super-enhancers, the regulatory regions driving cancer phenotype through epigenetic reprogramming. Indeed, adenocarcinoma super-enhancers classified according to their somatic alteration status display distinct epigenetic, transcriptional and pathway enrichments and are enriched in genetic risk SNPs associated with cancer predisposition [45].

#### 3.2.3. Epigenetic Targets for Cancer Therapy 

Unfortunately, the current cancer drugs targeting epigenetic mechanisms are unspecific and can often have serious side effects. Understanding other epigenetic changes in cancer is therefore highly urgent to open up avenues for new therapies. The pharmaceutical industry is therefore focused on identifying new compounds that target the reader, writer, and eraser mechanisms of histone modifications. To this end, functional genomics studies in disease are gaining pace. A recent large study generated ATAC-seq data, a proxy for mapping genome-wide open chromatin, in over 400 tumors across 23 cancer types from The Cancer Genome Atlas project [43]. The authors further identified enhancer–promoter interactions in different cancer types by integrating it with RNA-seq data and validated some of their predictions through CRISPR-Cas9 assays [43].

### 3.3. Largescale Epigenetic Studies in Other Diseases

The potential of epigenetic therapies for cancer treatment has influenced an increase in studies investigating epigenetic control across a wide range of other diseases. Such efforts have generated knowledge about the combinatorial effects of genetic mutations and epigenetics on the phenotype. For example, the interaction of genetic variants and DNA methylation of the interleukin-4 receptor gene increases the risk of asthma [62], and a genetic/epigenetic interaction in the reduced folate carrier (RFC1) gene locus influence fetal predisposition to autism [63]. The study of epigenetic mechanisms is highly relevant to some diseases. One of the major concerns of the aging world population today are neurodegenerative disorders. There is no cure for many of the neuropathies and the majority of the cases have no genetic basis. Many compounds function via epigenetic mechanisms, and epidrugs (discussed above) developed for cancer treatment have been submitted to clinical trials for the treatment of Alzheimer’s and Parkinson’s diseases [64]. For example, HDAC inhibitors change the epigenetic state and expression of *FXN* in the neurodegenerative disease Friedreich ataxia, making it highly effective in an in vitro disease model and also showing promising results in a patient study [65]. In summary, understanding of epigenomic landscape of neurodegenerative and other disorders will likely provide a possibility of early detection and intervention of pre-symptomatic pathological events. This will allow development and implementation of novel strategies or treatments to halt pathological progress. It is important to stress that it is the putative reversibility of epigenetic aberrations that enables pharmacological interventions (epidrugs) as potential novel candidates for successful treatments of multifactorial disorders [64].

## 4. Computational Approaches towards Epigenetic Data Analysis and Integration

### 4.1. Epigenetic Data Integration to Understand the “Epigenetic Code”

Several studies have connected specific combinations of histone modifications and DNA methylation to the presence or absence of transcriptional activity and genomic functional elements. For instance, H3K4me3 is highly enriched at the promoters of actively transcribed genes [25], H3K36me3 is found on the gene body of genes under transcription and high levels of H3K9me3 are associated with facultative heterochromatin [23]. ChIP sequencing technology has allowed to generate a genome-wide high-resolution map of the distribution and co-localization of histone marks. Large initiatives have focused on unravelling the human epigenetic landscape. The Roadmap Epigenomics consortium has collected 111 reference human epigenomes by profiling histone modification patterns, DNA accessibility, DNA methylation, and RNA expression to define global maps of regulatory elements, regulatory modules of coordinated activity, and their likely activators and repressors [41]. They further used a method based on Hidden Markov Models (HMMs) to derive a minimal informative set of epigenetic modifications for differentiating between cell types, tissues and development stages, as well as between healthy and diseased cells. Increasingly, epigenetic data is generated in clinical settings, for a move towards precision medicine. For example, Polak et al. [66] were able to pinpoint differences in the mutational landscape between cancers based on their cell type of origin. In their work, a random forest based approach was used to predict mutation densities using 424 predictor variables. When gene expression is available, together with DNA methylation levels and genotypes, one could construct a network of interactions between these features, as introduced by Hou et al. [67]. Such an approach is useful in prognosis of various cancers. This was also demonstrated by Zhu et al. [68], who tested a kernel machine learning method on various omics data and clinical factors to predict prognosis in 14 cancer types. They found that the prognostic power of copy number and somatic mutations was quite low compared to expression profiles. Moreover, they demonstrated that incorporating omics data to predictions based on clinical variables can improve the results, as it may account for the absence of unknown or unmeasured clinical features.

#### The Function of Epigenetic Modifications Still Remains Understudied 

Sekhon et al. [69] integrated five different histone modification datasets to predict gene expression levels with the use of deep neural networks. Hlady et al. [51] performed integrative analysis of multiple epigenetic modifications in hepatic cancer to identify epigenetic driver loci, and further demonstrated that two loci, *COMT* and *FMO3*, increase apoptosis and decrease cell viability in a liver-derived cancer cell line. There is an effort to integrate more and more epigenetic phenomena in such studies, but the large number of histone modifications possible at histone tails increases the combinatorial complexity of the histone code. Furthermore, histone modifications or the histone status varies during development [70]. The histone code is therefore complex and dynamic. More importantly, the causal relationship between histone modifications and transcription activity has not yet been deciphered. For example, H3K4me1 is present at regulatory elements called enhancers, and is widely used to predict enhancer elements [71]. However, whether H3K4me1 controls or simply correlates with enhancer activity and function has remained unclear. Recent studies suggest that H3K4me1 might fine-tune, rather than tightly control, enhancer activity and function [72].

### 4.2. Linking Epigenetic Mechanisms to Phenotypes: Epigenetic Epidemiology

#### 4.2.1. More Data Equals More Challenges 

The success of genome-wide association studies (GWAS) in identifying genetic loci associated with common diseases have facilitated exploration of epigenetic loci associated with diseases, also known as the epigenome-wide association studies (EWAS). Much focus in the EWAS-type analysis has been on genome-wide DNA methylation studies, where a statistical framework is developed to identify statistically significant association between the methylation level of each CpG site and the trait of interest (reviewed in References [73,74]). However, as the technologies constantly improve to make data from other epigenetic markers available, more and more researchers integrate this data, together with genetic information to improve predictions and risk assessment [75,76,77,78]. The integration of data from diverse sources is generally a daunting task. This challenge can be simplified with the help of new experimental methods such as assay for transposase-accessible chromatin using sequencing (ATAC-seq) allow for extracting information about different epigenetic phenomena from a single experiment [79]. Moreover, one can use existing databases that enable visualization of publicly available datasets, sometimes also giving the possibility to overlay user’s data [80].

#### 4.2.2. New Data Integration Opportunities 

The most widely applied method in epigenetic epidemiology is to use a regression model to check associations between variations in the data and the trait, as in standard epidemiology. This methodology is used by various studies where principal components (PCs) [81], level of methylation [82,83], or association score from EWAS analysis [84] are used to represent the variation. In order to facilitate the interpretation of the results from such an analysis, one typically uses bioinformatics databases to search for possible biological explanations for connections between the significant genomic regions and the trait of interest. This can be done, for example, in the Cistrome database [85] that gathers published gene regulatory data, and enables interactive visual analysis. Another easy-to-use tool is HaploReg [86]. Although the output is less intuitive, the database provides rich information about possible regulatory functions of SNPs or genomic regions of interest. Having found a set of genes that contain differential epigenetic modifications allows to perform a gene enrichment analysis, for example, with the help of LAGO (https://go.princeton.edu/cgi-bin/LAGO), STRING [87] or Reactome [88]. Another interesting possibility is to infer disease–gene connections by accounting for associations between different types of data, as implemented in Hetionet [89]. This tool integrates around 30 different databases, creating a heterogeneous network from information such as expression data, differential gene regulation, GWAS gene-trait associations, drug banks, etc. The implementation of the database in a neo4j network service allows for a quick online querying and visually appealing output that can inform on hidden connections between, for example, influence of vitamin intake, genes, and a disease [90].

#### 4.2.3. Epigenome-Wide Association Studies Analyses Are Informative Only about an Association and Not Causality 

In classical epidemiology Mendelian randomization (MR) is widely used to infer causality whenever a standard randomized trial is impossible to perform. It is based on an assumption that the underlying genotype is randomly assigned to each individual and is the cause of the measured exposure (e.g., body mass index (BMI)), not vice versa. This method has been recently adapted to DNA methylation data [91,92]. However, since DNA methylation can be both an inducer and the outcome of the disease, MR with epigenetic data needs to be used with caution [93]. Nevertheless, the remarkably simple idea behind the MR allows researchers to make very interesting claims, studying causality between the epigenetic marks and a wide range of outcomes, from blood lipid levels [94] through features such as physical aggression [95]. Used together with EWAS and GWAS analyses, MR gives us the possibility to propose biomarker loci or targets for therapies for patients [82].

Many more methods have been developed recently to infer causality from epigenetic data. For example, Howey et al. [96] fit a Bayesian network to the most significant findings from their linear regression modeling to show the directions of influence between DNA methylation and blood lipid levels. In another study, structural equation modeling (SEM) was used to search for the pathways by which the genetic variants lead to a disease [97]. With this method, one can establish significant interactions between all the different measurements (here, blood lipid levels, variant allele in the chosen SNP, and methylation levels on the nearby CpGs) and importantly, the model predicts the directionality of these interactions.

#### 4.2.4. Causality Inference from Translational Studies 

To test whether a change in the levels of epigenetic modifications is the cause or consequence of a disease, one can conduct a translational study, following patients over a specific time. Such time-dependent information can then be used to check whether a certain locus displays epigenetic changes, e.g., DNA methylation, before or after a certain event; disease onset. Using this concept, a computational approach GATE [98] has been implemented as a two-layer model, where one layer categorizes the spatial characteristics of the chromatin, and the other layer focuses on transitions between different chromatin states. This allows to create a model of transitions between different epigenetic states of a cell. Another recent method, ChromTime, uses the raw signal from data generated by CHiP-seq and similar techniques to track temporal changes in the peaks [99]. It not only detects diminishing or appearing peaks, but also asymmetrical changes in peak shapes. The authors further demonstrate that ChromTime can be applied on ATAC-seq, CHiP-seq, and DNase-seq data to infer on gene expression levels and TF binding.

### 4.3. Combining Levels of Epigenetic Marks within Genomic Regions 

One of the important shortcomings of many methods is that they consider each epigenetic locus independently of other loci to evaluate its significance for association with a certain trait. For example, the majority of studies focus on methylation level of one CpG at a time even when they integrate it with several other data sources. Recent studies summarized methylation level within a region [81,83], though this is not yet widely used despite the fact that changes in DNA methylation of only one CpG site would likely not lead to big changes in TF binding affinity to this site, unless it is followed by coordinated changes on neighboring CpG sites [100]. To this end, we developed a statistical framework that integrates DNA methylation and genetic information to identify statistically significant interactions between an SNP and methylation level within a group of neighboring CpGs [101]. The CpGs are grouped based on whether the CpGs are assigned to a promoter, enhancer or a gene body, to facilitate the downstream analysis for the biological interpretations.

**Figure 2:**
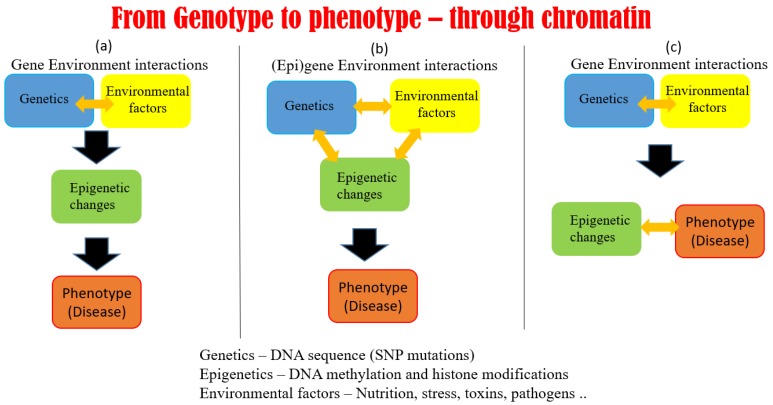
The figure depicts three likely scenarios where epigenetics might fit with from the genotype to phenotype (gene expression) information flow: (**a**) epigenetic changes are downstream of gene environment interactions and determine the phenotype; (**b**) genome sequence, environment, and epigenetic modification work together to establish the phenotype; and (**c**) epigenetic landscape and phenotype are both determined and established by gene–environment interactions. SNP: single nucleotide variations

The ultimate goal is to understand how genetic and epigenetic variations manifest in a phenotype under certain environmental conditions (Figure 2). To this end, an ideal computational approach would take into account the genotype and several epigenetic modifications at the same time, to explain a phenotype or perhaps a proxy such as transcriptomic data. There is already a huge amount of such data in the public domain, and tools and resources such as Omics Discovery Index web service (https://www.omicsdi.org/) to search for datasets. There is a need to take the advantage of this enormous amount of data, test ideas, and to develop tools to maximize the information extraction from the data.

## 5. Conclusions

### 5.1. Possible Scenarios Linking Epigenetics, Genetics, and Phenotype 

Hundreds of human cell types have a unique gene expression signature despite sharing the same genome sequence, largely due to tight control by epigenetic modifications of the non-coding genome in a cell type specific manner. Epigenetic aberrations are thought to result in complex diseases such as cancers. The vast majority of genetic variants found associated with common diseases by genome-wide association studies are indeed located in the non-coding genome. Only in a very limited number of cases such as for Crohn’s disease or rheumatoid arthritis, have these associations led to the successful identification of causal genes with a potential of being therapeutic targets. However, most disease-associated variants have no known biological context to disease, limiting their utility for prognosis or treatment. Human epidemiological studies provide evidence for prenatal and early postnatal environmental factors influencing adult risk of developing various chronic diseases, such as cancer, cardiovascular disease, diabetes, obesity, and behavioral disorders such as schizophrenia [17]. Some of these environmental factors can be linked directly to alterations of the epigenetic landscape that affect gene regulation and finally the disease. Though the association is proven in many cases, the chain of causality remains to be established. This leads to three possible scenarios of how epigenetic mechanisms control genes and influence disease occurrence (Figure 2). The first scenario is where environmental factors alter epigenetic modifications, which in turn alter the phenotype (Figure 2A). This scenario is supported by mouse experiments where maternal methyl-donor supplementation during pregnancy with folic acid, vitamin B12, choline, and betaine was shown to affect the phenotype of the Avy (viable yellow agouti) offspring by directly altering the epigenome [102]. The second possibility is that gene–environment interactions affect both epigenetic status and transcription read-out, as their correlation does not imply causality (Figure 2B). Indeed, as most of the epigenetic modifications are “lost” during the gametogenesis, this scenario is assumed to be true for many cases. Careful research has nevertheless identified that at least some epigenetic modifications are passed on to the next generation [103]. This leads to a third scenario where epigenetic modifications are not downstream of but work together with gene environment interactions to result in a phenotype (Figure 2C). The relative abundance of the three scenarios and the molecular mechanisms controlling them need to be understood. Over the coming years, research should be focused not only on identifying epigenetic phenomena affecting gene regulation to find epigenetic biomarkers for disease and environmental exposure, but also on establishing the causal relationship between the three components (gene–environment, epigenetics, and phenotype). Only by understanding causal relations can we develop new epigenetic interventions to truly revolutionize medicine to move towards preventive medicine.

### 5.2. New Approaches and Technologies Must Aim on Establishing a Causal Link between Epigenetics and Disease 

The most important challenge in precision medicine is thus to link genetic variation within the non-coding genome to candidate causal gene(s) or pathways for disease or other physiological phenotypes. It is urgent not only to identify the regulatory regions but also the spatial organization of DNA to understand how these regulatory regions interact to manifest into a phenotype. It is now accepted that a large number of possible regulatory interactions are potentially pathogenic and might be unique to tumors [43]. Although experimental techniques such as chromosome conformation capture (3C, 4C) [104] combined with next generation sequencing (Hi-C) show a great promise [105,106], their time and cost will limit the availability of comprehensive, experimentally verified 3D chromatin landscapes to a tiny fraction of the hundreds of different human cell types in the foreseeable future. The development of novel cost-effective high-throughput experimental methods is ongoing. Meanwhile, computational tools to predict enhancer–promoter interactions will be essential to model the effects of non-coding genetic variation on epigenetic modifications and downstream gene expression programs in human health and disease. Though a regulatory region is associated to its proximal promoter, the integration of known or putative enhancer promoter interactions in GWAS analysis has a potential to identify novel disease associated genes and pathways [107]. This will require a significant leap beyond studies which have only used correlations between epigenetic states of enhancers with promoter expression [108]. We have recently performed preliminary work to establish causality using regulatory information [109]. More computational approaches to systematically combine epigenetic information into causal network models are needed.

### 5.3. Epigenetic Studies and Therapies Have an Important Role in Shaping the Future of Medicine

Finally, segregating patients based on different factors into more coherent groups for better treatment is the foundation of precision medicine, but many factors used to stratify patients have no known functional mechanisms. For example, sexual differences in cancer risk and survival are well studied, with males having an increased risk and poorer survival for most cancers [110]. The understanding of functional mechanisms behind these sex differences is gathering pace. For example, male breast cancer is rare, poorly characterized and resistant to hormonal treatment. An integrative epigenetic and transcriptomic analysis revealed a gender-selective and genomic location-specific hormone receptor action associated with survival in male breast cancer [39]. Epigenetics therefore has a big role to play in the foundations of the precision medicine.

## Figures and Tables

**Table 1:** A collection of epigenetic studies (excluding DNA methylation) in disease context including the data type, number of samples, disease type, and publication reference.

Num.	Data Type	Disease	Available data	# of Samples	Reference
1	ATAC-seq	23 cancer types	Genotype, ATAC-seq, RNA-seq	410	[43]
2	ChIP-seq	Prostate cancer	H3K27ac, H3K4me3, H3K27me3	100	GSE120738
3	ChIP-seq	Breast cancer	H3K4me1, TFs	-	[44]
4	ChIP-seq	Adenocarcinoma	H3K27ac, H3K4me3, H3K4me1	94	[45]
5	ChIP-seq	Acute myeloid leukemia	H3K9me3	108	[46]
6	ChIP-seq	Glioma	Multiple	-	[47]
7	ChIP-on-chip	Acute myeloid leukemia	H3	73	[48]
8	ChIP-on-chip	Acute promyelocytic leukemia	H3, H3K9me3, H3K4me3	372	[49]
9	ChIP-seq	Acute myeloid leukemia	H3K9me2	16	[50]
10	ChIP-seq	Hepatocarcinoma	Multiple	5	[51]
11	ATAC-seq, ChIP-seq	Colorectal cancer	Multiple	4	[52]
12	FAIRE-seq, ChIP-seq	Ovarian cancer	H3K27ac, H3K4me1	5	[53]
